# Indoor ozone removal and deposition using unactivated solid and liquid coffee

**DOI:** 10.1371/journal.pone.0273188

**Published:** 2022-08-16

**Authors:** En-Ying Jiang, Tsrong-Yi Wen

**Affiliations:** 1 Department of Mechanical Engineering, National Taiwan University of Science and Technology, Taipei, Taiwan; 2 High Speed 3D Printing Research Center, National Taiwan University of Science and Technology, Taipei, Taiwan; Tsinghua University, CHINA

## Abstract

Managing indoor ozone levels is important because ozone is a hazardous pollutant that has adverse effects on human health. Coffee is a popular daily beverage, and thus, coffee beans and spent coffee grounds are common in many places such as offices, homes, aircraft, cafeterias, and such. The most common material used to remove ozone is activated carbon which can be made from coffee beans or spent coffee grounds with proper activation processes. This paper presents a novel idea: to remove ozone at the level of an indoor environment using unactivated coffee products. This paper examines the ozone removal efficiency and the ozone deposition velocity at 130 ppb ozone for two types of coffee: solid coffee (powder) and liquid coffee (beverage). The activated carbon, the deionized water, and the seawater are also included for comparison and validation purposes. The tests show that the fine coffee powder has a removal efficiency of 58.5% and a deposition velocity of 0.62 cm/s. The liquid coffee has a removal efficiency of 34.4% and a deposition velocity of 0.23 cm/s. The chemical inspections indicate that the oxidation reactions with the carbohydrates in solid coffee and the metal/mineral elements in liquid coffee are responsible for ozone removal. These results have confirmed that ozone removal via coffee is effective, controlling indoor air quality by coffee products is thus becoming possible.

## Introduction

**Fig 1:**
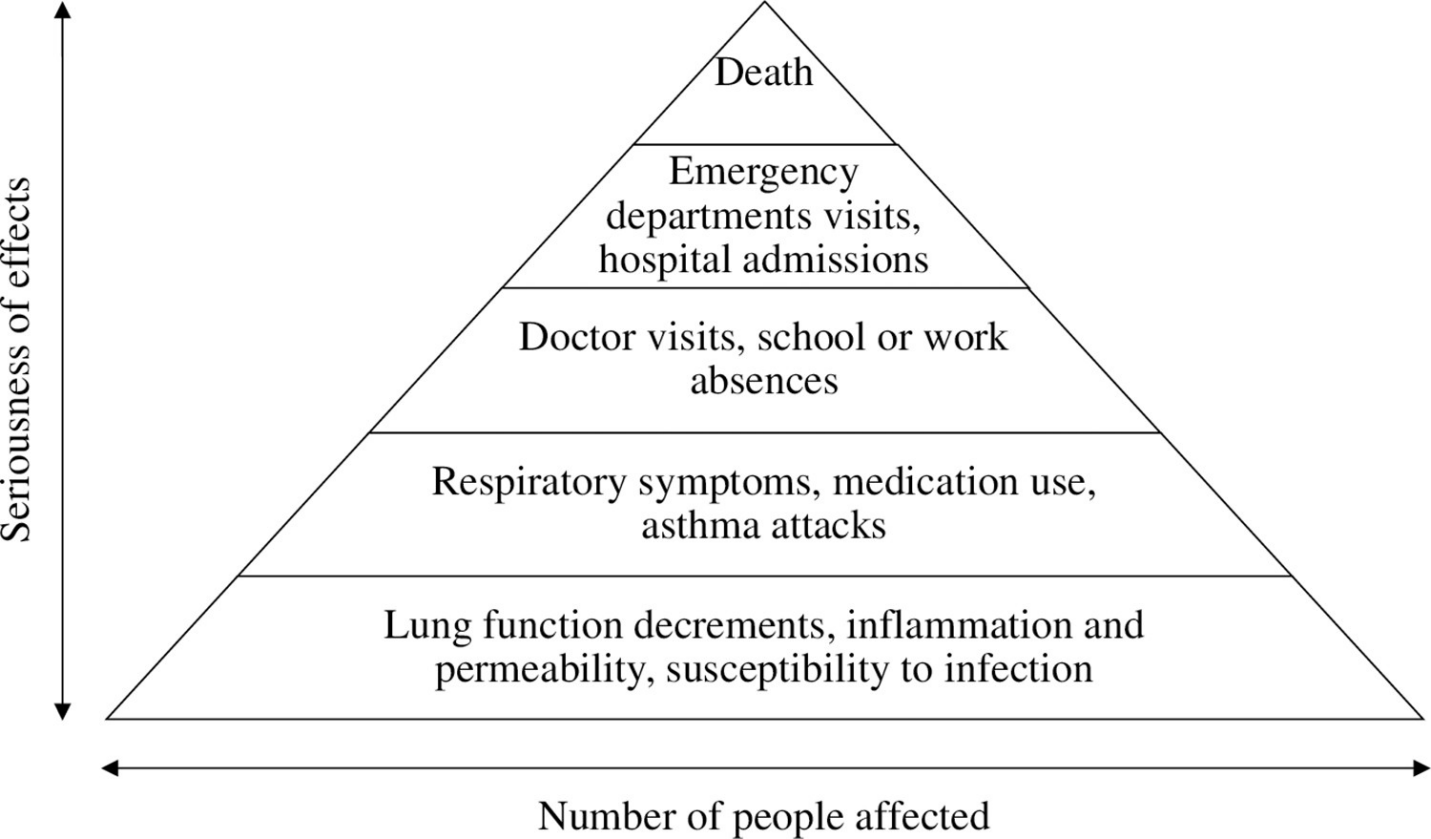
The effects of ozone on human health [1].

Ozone consists of three oxygen atoms, smells irritating at room temperature, and is one of the six principal air pollutants defined by the U.S. Environmental Protection Agency (EPA). Ozone is toxic to humans so that exposure to a high ozone environment could cause symptoms and diseases like cough, dry eyes/skin, headache, asthma, cardiovascular diseases, mortality, and more [1–3], as shown in Fig 1. Furthermore, the U.S. EPA correlates the air quality index (AQI) with the ozone level and suggests that the significant harm level for ozone is 600 ppb (2-hour average), as shown in **Table 1** [4]. Therefore, managing indoor ozone levels has significance from the public health point of view.

**Table 1:** The category of the air quality index and the ozone concentration breakpoints [4].

AQI Category	Index Values	Ozone Breakpoints (ppb, 8-hour average)
Good	0 to 50	0 to 54
Moderate	51 to 100	55 to 70
Unhealthy for Sensitive Group	101 to 150	71 to 85
Unhealthy	151 to 200	86 to 105
Very Unhealthy	201 to 300	106 to 200
Hazardous	301 to 500	201 to Significant Harm Level

Ozone can be produced on purpose (e.g., discharging process [5]) or in natural (e.g., the reaction of oxygen and ultraviolet light [6, 7]). The sources of indoor ozone are various, including common office/home electronics and outdoor ozone. A regular laser printer can emit ozone by 1.0 μg/copy to 1.2 μg/copy [8], while the ozone concentration of a copying room can be up to 146 ppb [9]. A commercial electrostatic air cleaner can generate ozone at a rate of 2.9 mg/hr and make the ozone face emission concentration of 44 ppb [10]. Besides, indoor ozone depends on outdoor ozone as well. The ratio of indoor ozone to outdoor ozone can be as low as 0.1, indicating that indoor ozone can be predominantly driven by outdoor ozone [11]. On the other hand, in the cabin of a cruising aircraft (where the outdoor ozone level is 500 ppb to 800 ppb [12]), the ozone concentration can be up to 250 ppb without a proper ozone converter [13]. Thus, taking control of the indoor ozone level is important and necessary for people’s health and comfort.

Activated carbon (AC) is the most common material used to remove ozone because of its porous structure and organic nature, while AC can be made from carbonaceous materials such as pinewood [14], straw [15], and solid coffee [16]. It is certain that having AC-based catalytic ozone converters (e.g., Pd-AC [17] and TiO_2_-AC [18]) or appropriate ventilation/filtration systems can lower the indoor ozone level [19–21]. Other than removing ozone actively, ozone can be removed passively by depositing onto materials such as carpet, fabric, plastic, human skin, and spent coffee grounds [22–24]. Coffee is one of the most common daily beverages in this modern society, if the ozone removal capability of coffee products is effective, coffee products could be further developed for indoor ozone removal or even other air pollution control purposes. Yet, no literature has reported the ozone removal capability (including removal efficiency and deposition velocity) of both solid coffee and liquid coffee at an indoor ozone level.

This paper presents the ozone removal efficiency and the ozone deposition velocity of both solid coffee and liquid coffee at an indoor ozone level. For the solid coffee (fresh and unused), two coffee powder sizes and one coffee bean were examined. The deionized (DI) water, the activated carbon, and the seawater were also tested for comparison and validation purposes. The inspections were carried out to justify the insights on what ozone reacted with the materials under test, including energy-dispersive X-ray spectroscopy (EDX), X-ray photoelectron spectroscopy (XPS), and inductively coupled plasma mass spectrometry (ICP-MS).

## Material and methods

### Experimental setup and procedures

**Fig 2:**
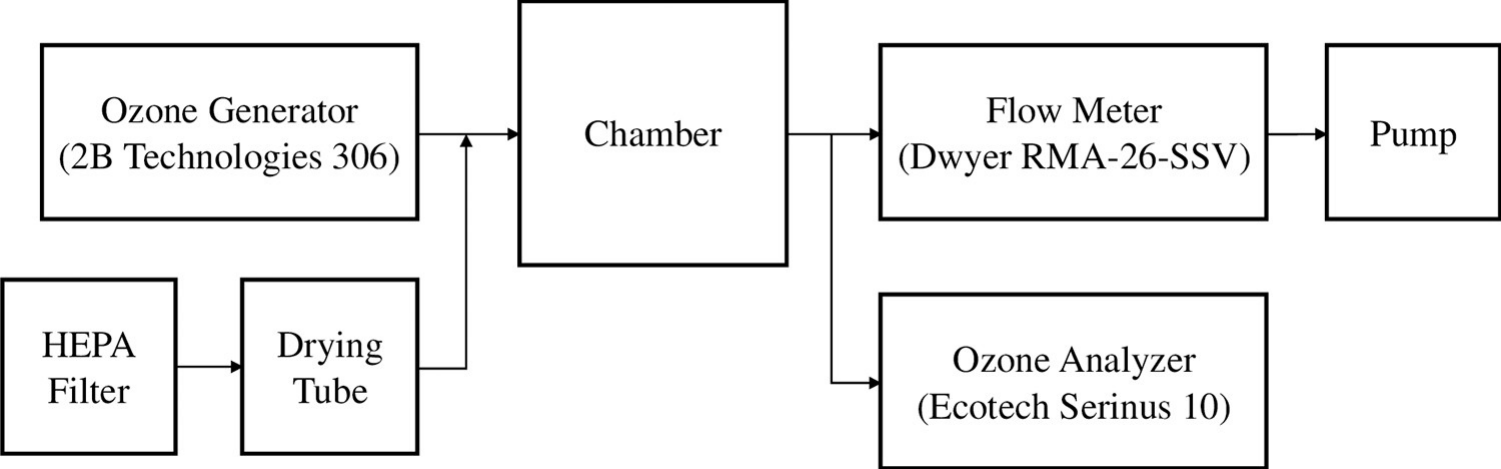
The schematic of the experimental setup.

Fig 2 shows the schematic of the experimental setup. The test was conducted in a 45 cm cubic stainless-steel chamber that was acting as an indoor environment. A specific opening with a sealing lid was made on the chamber door so that the material under test can be slid into the bottom of the chamber. The ozone generator was used to supply ozone to the chamber, and the fresh air entering the chamber was properly filtered and dried. The air exchange rate of the chamber was set to be 0.5 h^-1^ by controlling the pump and the flow meter connected downstream of the chamber. The entire setup was placed in a lab environment at an average temperature of 24°C ± 1.6°C, average humidity of 61.9% ± 6.9%, and one atmospheric pressure.

Before the test started, the chamber was cleaned thoroughly using ethanol and DI water. The ozone analyzer monitored the ozone concentration of the chamber nonstop for 16 hours and 10 minutes. The chamber was empty for the first eight hours to quench the chamber (passivate reactive sites) and to make sure the ozone concentration in the chamber was steady at 130 ppb. A glass container loaded with the material under test was then slid into the bottom center of the chamber through the designed opening exactly at the eighth hour. The ozone concentration of the chamber was kept monitored for another eight hours and 10 minutes. To ensure repeatability, five tests were conducted for every testing leg. Note that the glass container was used because glass has been proved to be not ozone reactive [25, 26].

### Materials under test

**Fig 3:**
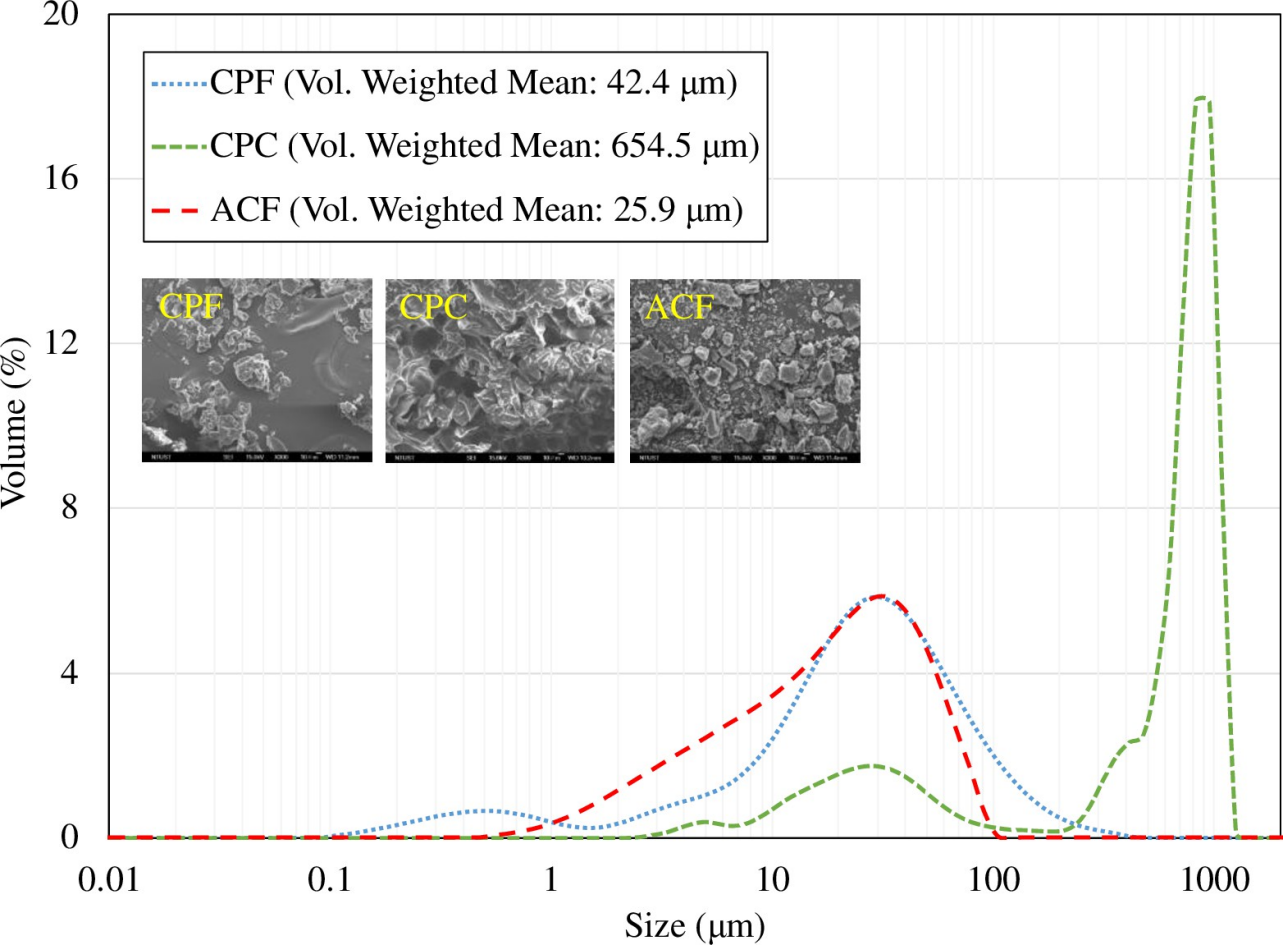
The size distribution and the SEM photos of the fine/coarse coffee powder and the activated carbon powder.

**Table 2** summarizes the information of all the materials under test. The coffee powder was made by grinding the fresh and unused coffee beans using a commercial grinder (Cuisinart DBM-8TW) at different size settings. After grinding, the coffee powder was sieved. The commercial activated carbon powder was also sieved to ensure size distribution. Fig 3 shows the size distributions along with the photos of the coffee powder and the activated carbon powder using a particle size analyzer (Malvern Mastersizer 2000) and a scanning electron microscope (SEM, JEOL JSM-6500F).

**Table 2:** The information of the materials under test, where *d*_p_ is the volume weighted mean size.

ID	Material	Physical Type	Description
CPF	Coffee (Starbucks, Breakfast Blend)	Solid	Fine Powder (*d*_p_ = 42.4 μm)
CPC	Coffee (Starbucks, Breakfast Blend)	Solid	Coarse Powder (*d*_p_ = 654.5 μm)
CB	Coffee (Starbucks, Breakfast Blend)	Solid	Raw Bean
ACF	Activated Carbon (Emperor Chemical Co Ltd)	Solid	Fine Powder (*d*_p_ = 25.9 μm)
CL	Coffee (Starbucks, Breakfast Blend)	Liquid	1035 ml H_2_O + 50 g CPC
SW	Seawater	Liquid	8.75 g sea salt + 250 ml DI water

The liquid coffee was made by a commercial coffee machine (Frigidaire FKC1151HS) using the daily and average coffee-making procedures at a 15-minute brewing time. The seawater was made by mixing 8.75 g of sea salt and 250 ml of deionized water so that the salinity was 35 g/kg (same as the real seawater).

A 1.3-cm depth and 6-cm diameter glass container was used to carry the materials under test. For all the powder materials, the amount loaded was 2-mm thick in the container; for the coffee bean, the amount loaded was 10 g; for all liquid materials, the amount loaded was 10 ml.

### Removal efficiency and deposition velocity

The ozone uptake by a material is dominated by two sequences: 1) ozone transportation to the material surface and then 2) the reaction between ozone and the material surface [27]. This paper uses the ozone removal efficiency (system dependent) and the ozone deposition velocity (material dependent) to evaluate the ozone removal performance of the materials under test, as shown in (1) and (2), respectively [28, 29].

$$\eta =1-\frac{C_{\mathrm{outlet}}}{C_{\mathrm{inlet}}}$$ (1)

$$v_{d}=\frac{Q(C_{\mathrm{in}}-C_{\mathrm{ss}})}{AC_{\mathrm{ss}}}$$ (2)

where *η* is the ozone removal efficiency, *C*_inlet_ is the initial ozone concentration, *C*_outlet_ is the outlet ozone concentration, *v_d_* is the ozone deposition velocity, *Q* is the airflow rate throughout the chamber, *C*_in_ is the ozone concentration entering the chamber, *C*_ss_ is the steady state ozone concentration of the chamber with the material under test, and *A* is the surface area of the glass container (loaded with the material under test). Note that the ozone concentration is represented by the last 2-hour average concentration.

## Results and discussion

### Ozone removal efficiency

**Fig 4:**
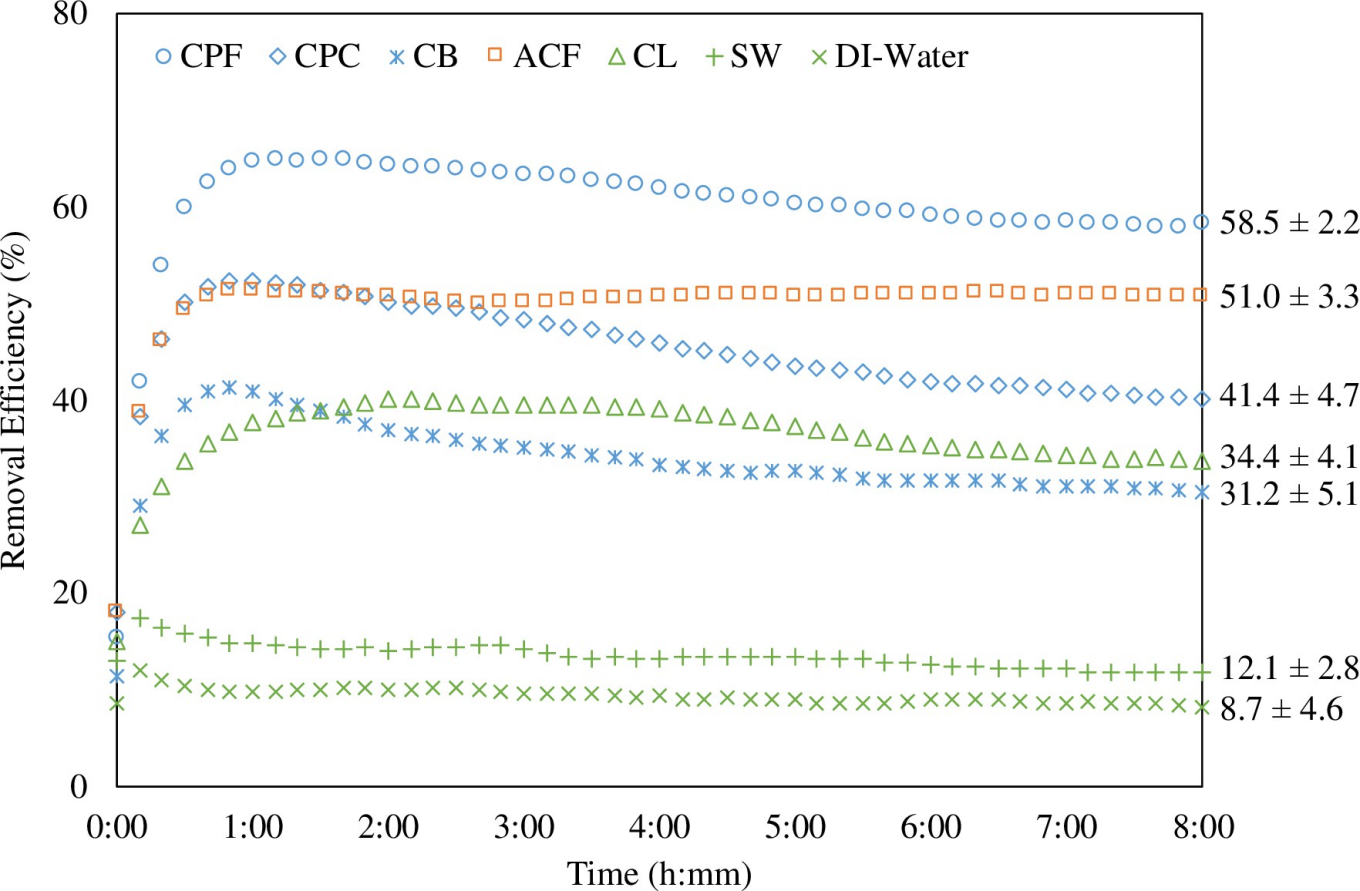
The ozone removal efficiency of the materials under test. Every data point represents a 10-minute average removal efficiency. The data point at 0:00 is an average from 0:00 to 0:10 and the data point at 8:00 is an average from 8:00 to 8:10. The ozone removal efficiency labeled on the right is the average removal efficiency from 6:00 to 8:00.

Fig 4 shows the ozone removal efficiency of all the materials under test. For the solid coffee, it shows that the ozone removal efficiency is physical size dependent. The fine and the coarse coffee powder have an ozone removal efficiency of 58.5% and 41.4%, respectively. The coffee bean has an ozone removal efficiency of 31.2%. Such a tendency is attributed to the fact that ozone removal is proportional to the surface area of the material [23]. Solid coffee is able to react with ozone because coffee contains various organic compounds (results are shown in the later section) that are known to be chemically oxygen affinity [30, 31]. On the other hand, the activated carbon powder also shows 51% ozone removal efficiency because of the large specific surface area, the organic nature, and the strong adsorption capacity [32, 33].

In general, the liquid materials have a lower ozone removal efficiency than the solid ones. This is because ozone removal is surface area dependent [23]. In this paper, the surface area of liquid material is simply the same as the surface area of the container, which is around 0.0028 m^2^. The surface area of a porous solid material can be obtained by the specific surface area. Consider one gram of a fresh coffee powder that has a specific surface area of 0.349 m^2^/g [23], the effective surface area is 0.349 m^2^, which is two-order larger than that of the counter liquid material. Moreover, the liquid coffee shows a competitive ozone removal efficiency to the coffee bean and significantly higher performance than the seawater and the DI-water. The reason is that liquid coffee contains several ozone-reacting metal and mineral elements (results are shown in the later section). Besides, despite it not being much, the DI-water and the seawater show a removal efficiency of 8.7% and 12.1%, respectively, simply because ozone is water soluble [34].

### Ozone deposition velocity

**Fig 5:**
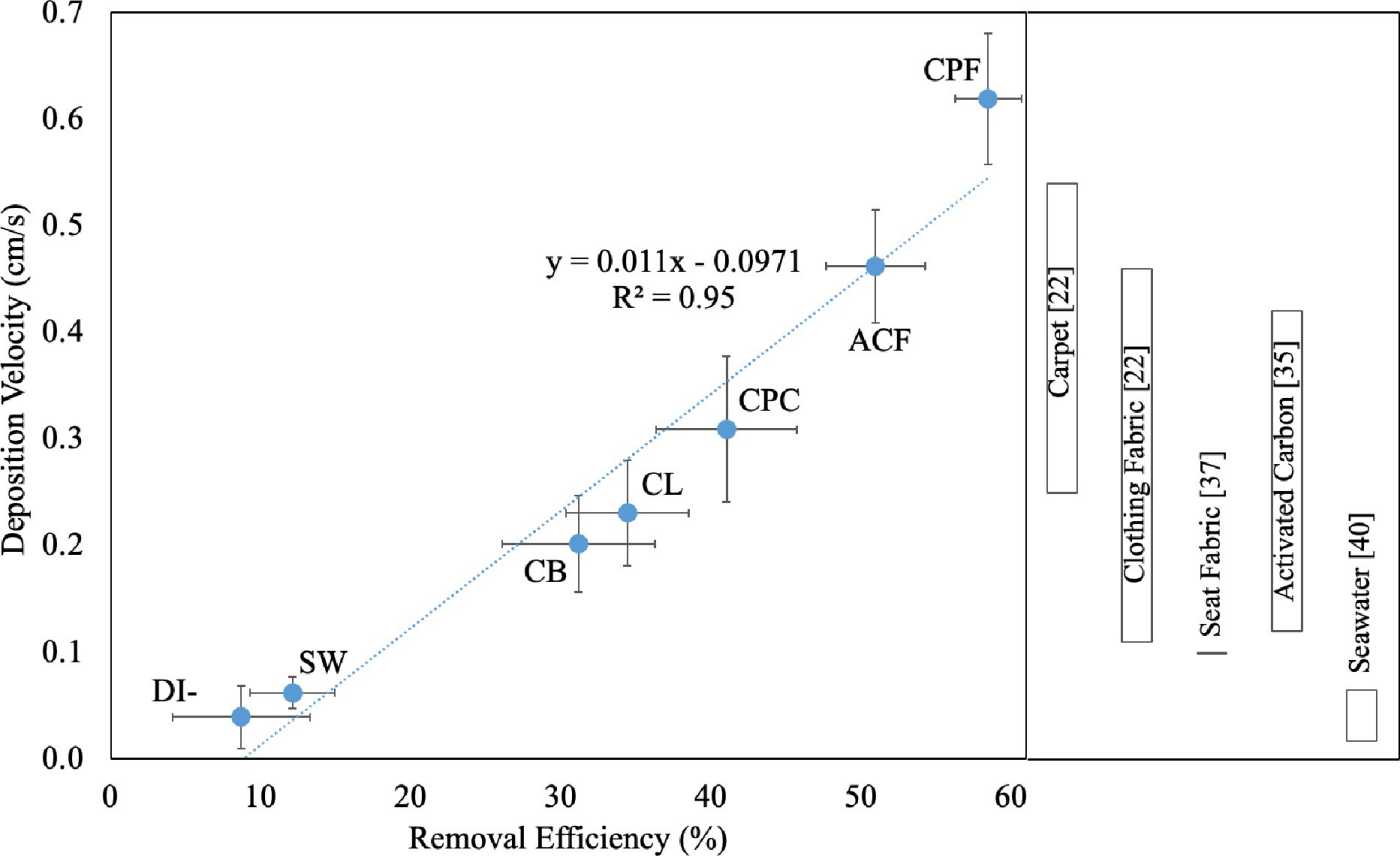
The deposition velocity versus the removal efficiency for the materials under test. The bars on the right are the deposition velocity ranges for the materials reported in the literature.

Fig 5 shows the ozone deposition velocity against the ozone removal efficiency. The deposition velocity of the activated carbon and the seawater is 0.46 cm/s and 0.062 cm/s, respectively, agreeing with those reported in the literature (0.12 cm/s to 0.42 cm/s for activated carbon [35] and 0.017 cm/s to 0.065 cm/s for seawater [36]). Furthermore, the deposition velocity is a close-to-linear function of the removal efficiency, suggesting that the experimental conditions (air exchange rate, ozone concentration, dimension/geometry of glass container, etc.) were well-controlled.

The deposition velocity of the solid materials is higher than that of the liquid ones because the removal efficiency has the same trend. The fine coffee powder exhibits the highest deposition velocity and is about twice and triple higher than the coarse powder and the coffee beans, respectively. Such results imply that the ozone removal by solid coffee has a strong dependency on the surface area of the material under test. Comparing with the data reported in the literature, the deposition velocity of the solid coffee perform similarly with or even outperform certain common indoor materials/objects such as carpet (0.25 cm/s to 0.54 cm/s [22]), clothing fabric (0.11 cm/s to 0.46 cm/s [22]), and seat fabric (0.10 cm/s [37]).

On the other hand, the deposition velocity of the liquid coffee is similar to that of the coffee beans and is much higher than that of both seawater and DI-water. This can be explained by that the coffee contains various carbohydrates [38, 39] such that the dissolved compounds along with various metal and mineral elements in the liquid coffee (results are shown in the later section) help the ozone removal/decomposition [40].

### Physical and chemical inspections

#### Element and surface analysis for solid materials under test

**Table 3** shows the EDX (JEOL JSM-6500F) results for the solid materials under test. It can be seen that, regardless of the material, the carbon decreases while the oxygen increases after reacting with ozone. Taking the oxygen-to-carbon (OC) ratio as an indicator of the level of oxidation [41, 42], the OC ratio change of the coarse coffee powder is lower than that of the fine coffee powder because the effective reacting surface area of the coarse coffee powder (thus the level of oxidation) is smaller than that of the fine coffee powder. On the other side, the OC ratio of the activated carbon powder increases significantly by +73.7% after reacting with ozone because of the large surface area and the release of the organics (the sign of oxidation) [43].

**Table 3:** The element analysis for solid materials under test before and after reacting with ozone.

Material	Ozone Reaction	Carbon (wt. %)	Oxygen (wt. %)	O: C
CPF	Before	68.1	27.9	0.41
	After	63.9	31.3	0.49
	Percent Change	− 6.2%	+ 12.2%	+ 19.5%
CPC	Before	65.6	31.7	0.48
	After	64.4	32.5	0.50
	Percent Change	− 1.8%	+ 2.5%	+ 4.2%
ACF	Before	96.3	3.7	0.038
	After	93.5	6.2	0.066
	Percent Change	− 2.9%	+ 67.6%	+ 73.7%

**Fig 6:**
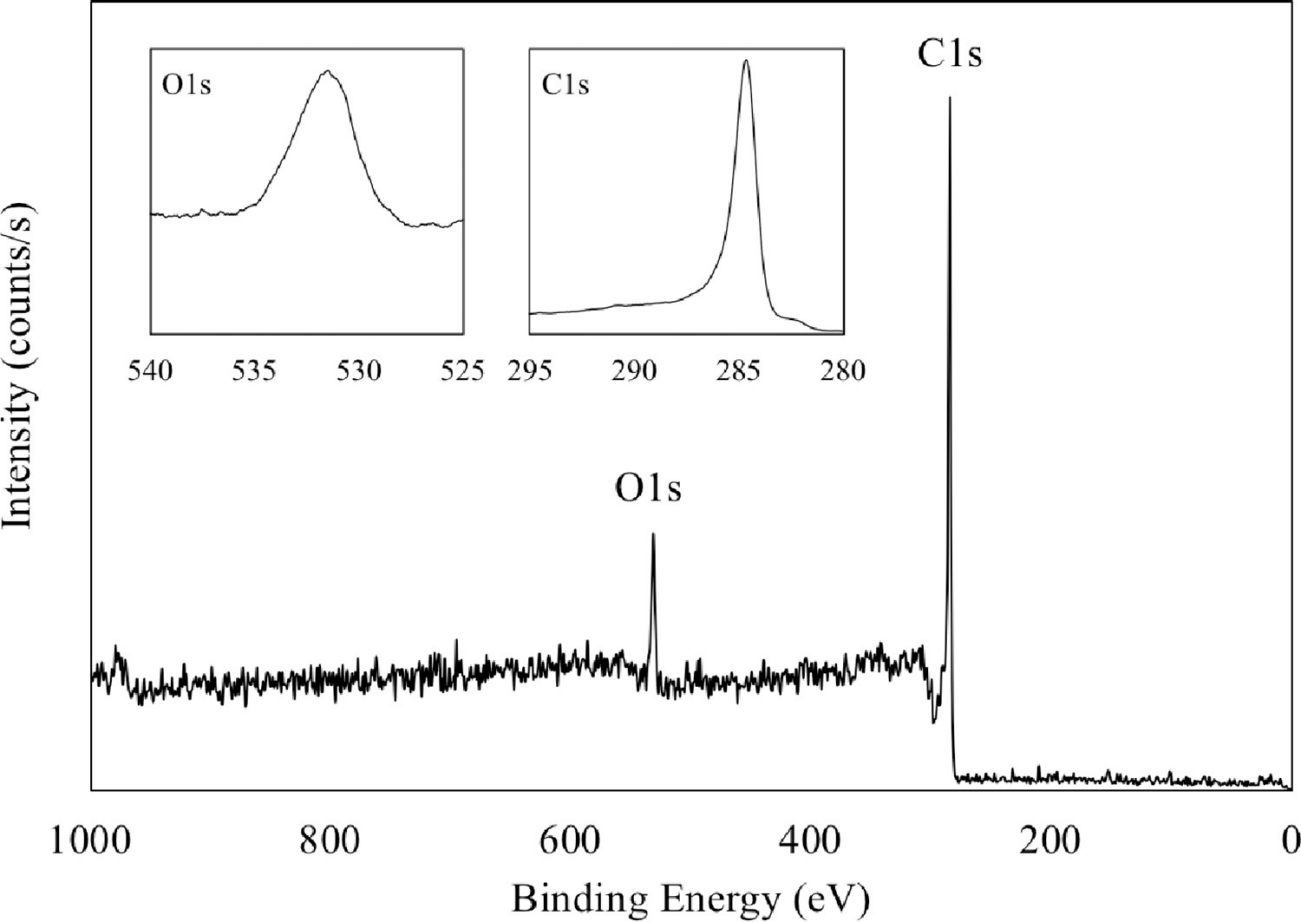
The survey spectrum of the coarse coffee powder.

**Fig 7:**
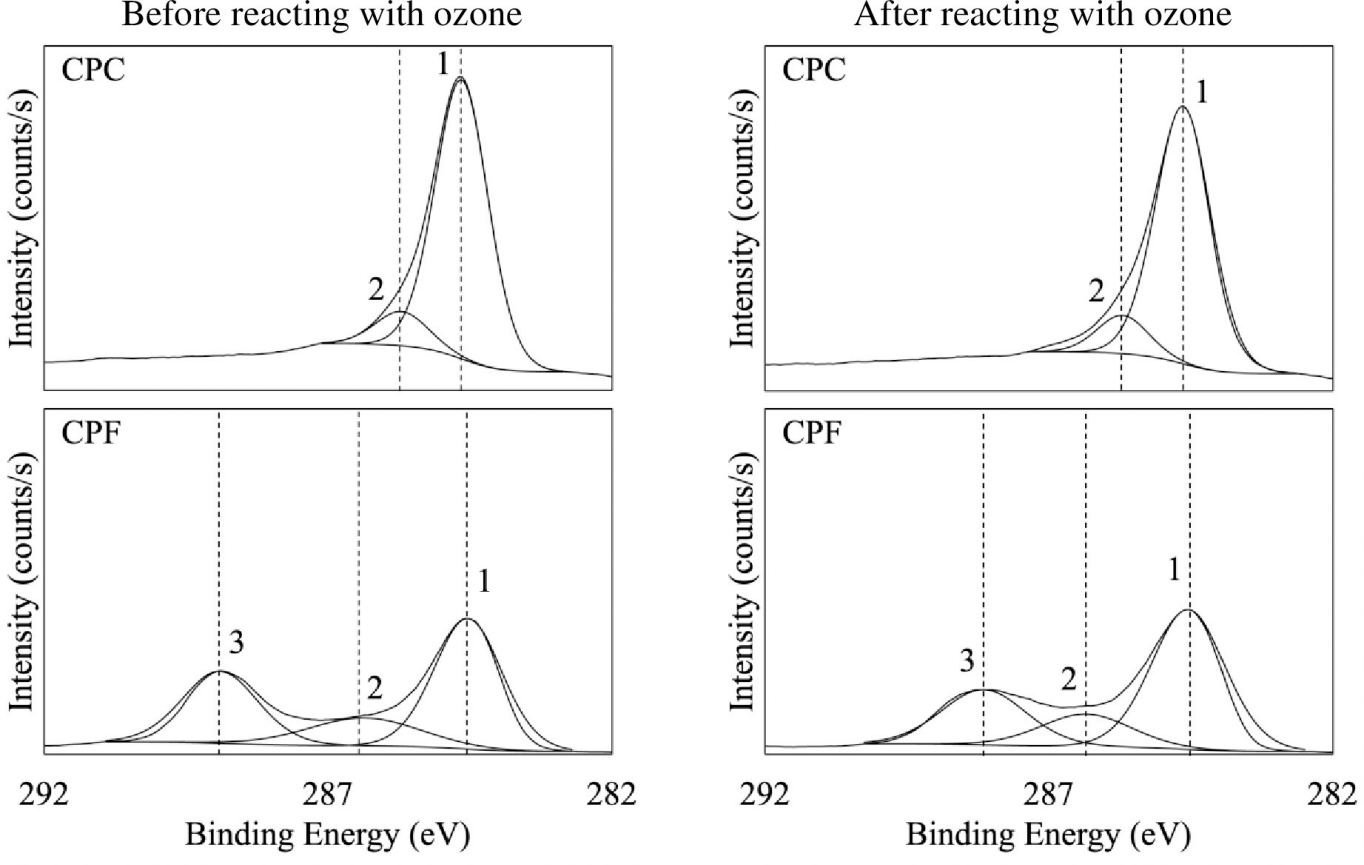
The XPS C1s core levels of the coffee powder before and after reacting with ozone.

The XPS analysis was conducted (Thermo Scientific, Theta Probe) to analyze the surface functional groups and the binding energy for the coffee powder. Fig 6 shows the survey spectrum of the coarse coffee powder and there are two clear peaks for C1s and O1s at 284.6 eV and 531.4 eV, respectively, close to the data reported [44]. Fig 7 and **Table 4** show the C1s core levels for both the fine and the coarse coffee powder that were conducted before and after reacting with ozone. It demonstrates that there are three oxidation-related functional groups, including C–C (graphitic carbon),–C–O–or C–OH (hydroxyl, phenolic, alcoholic, or etheric group), and C = O (carbonyl or quinone group). Fig 7 illustrates that the intensity of all these three functional groups decreases after reacting with ozone, which is an obvious and clear characteristic of oxidation. However, the major functional group responsible for the oxidation between the coarse and the fine coffee powder is different. For the coarse coffee powder, the C–C group takes the primary place for the ozone reaction (as shown in (3) [45]) as the intensity of the C–C group decreases more obviously than the–C–O–or C–OH group. On the other hand, for the fine coffee powder, the C = O group is responsible for the major oxidation because the intensity of the C = O goes down but the C–C group does not change much. This provides evidence that the fine coffee powder reacts with ozone stronger than the coarse coffee powder does because the C–OH and C = O are highly ozone reactive (as shown in (4) [46]). However, the accumulation of the–C–O–/C–OH/C = O groups could block the carbon surface and decrease the ozone decomposition rate [46], evident that the ozone removal efficiency of the solid coffee decreases over time (Fig 4).

**Table 4:** The XPS identified functional groups corresponding to Fig 7.

Peak	Functional Group(s)
1	Graphitic Carbon (C–C) [47, 48]
2	Hydroxyl, phenolic, alcoholic or etheric group (–C–O–, C–OH) [41, 47, 49]
3	Carbonyl or quinone group (C = O) [48, 49]


$$-C-C-+O_{3}\to -C-\mathrm{OOO}-C-$$ (3)

$$C_{n}\overset{O_{3}}{\to }(-C-\mathrm{OH},-C=O)+O_{2}$$ (4)

#### Element analysis for liquid coffee under test

As shown in **Table 5**, the ICP-MS results (PerkinElmer NexION 2000) reveal that the liquid coffee contains several metal elements and minerals, including magnesium, manganese, iron, calcium, potassium, and others, coinciding with the compositions reported in the literature [50–52]. The reaction between ozone and metals is essentially a redox reaction (reduction-oxidation). Ozone can decompose to hydroxyl radical OH^−^ and superoxide O_2_^−^, while the former is a strong oxidant to react with various substances [53, 54]. The literature presented that magnesium, manganese, and iron are able to react with ozone effectively, as shown in (5) [55]. Besides, there is a literature showing that the calcium in water helps ozone decomposition as well [56]. However, it was indicated that the ozone decomposition in water can be significantly low when there present hydroxyl radical scavengers [57], while caffeine is an effective hydroxyl radical scavenger (with a rate of 5.9×10^9^ 1/Ms) [58]. Overall, the metal and the mineral compositions in the liquid coffee along with the ozone solubility in the water contribute to ozone removal, but the caffeine somewhat counters such effectiveness. Thus, the ozone removal by the liquid coffee is not as good as that by the solid coffee.

**Table 5:** The element analysis for the liquid coffee under test.

Element	Mg	Mn	Fe	Ca	K	P	S
Concentration (ppm)	89.9	0.54	0.88	7.0	1893.9	50.4	38.0


$$M^{n+}+O_{3}+H^{+}\to M^{(n+1)+}+{\mathrm{HO}}^{\cdot }+O_{2}\;\mathrm{where}\;M\;\mathrm{represents}\;\mathrm{Mg},\;\mathrm{Mn},\;\mathrm{or}\;\mathrm{Fe}$$ (5)

## Conclusions

This paper presents the ozone removal efficiency and the ozone deposition velocity of the solid coffee (powder) and the liquid coffee (beverage). Other materials, including the activated carbon (powder), the seawater, and the DI-water, are covered as well for comparison and validation purposes.

The results show that the ozone removal efficiency and the ozone deposition velocity of the materials under test have a positive correlation. The ozone removal efficiency of the coffee powder is size dependent, i.e., finer powder has a higher ozone removal efficiency. The ozone removal efficiency and the ozone deposition velocity of the fine coffee powder are 58.5% and 0.62 cm/s, respectively, slightly higher than those of the activated carbon (51.0% and 0.46 cm/s). The liquid coffee exhibits a moderate ozone removal performance, a removal efficiency of 34.4% and a deposition velocity of 0.23 cm/s, higher than those of the deionized water (8.7% and 0.04 cm/s) and the seawater (12.1% and 0.06 cm/s). Furthermore, the chemical inspections suggest that the ozone removal by coffee is attributed to the oxidation processes because coffee is full of hydrocarbon compounds, metal elements, and mineral substances that are oxygen affinity. These results suggest that ozone removal by coffee products is effective and imply that using similar hydrocarbon materials for indoor air quality control purposes might also be possible.
